# Hepatic lysosomal acid lipase overexpression worsens hepatic inflammation in mice fed a Western diet

**DOI:** 10.1016/j.jlr.2021.100133

**Published:** 2021-10-05

**Authors:** Michael W. Lopresti, Wenqi Cui, Breann E. Abernathy, Gavin Fredrickson, Fanta Barrow, Arnav S. Desai, Xavier S. Revelo, Douglas G. Mashek

**Affiliations:** 1 Department of Biochemistry, Molecular Biology, and Biophysics, University of Minnesota, Minneapolis, MN, USA; 2 Department of Integrative Biology and Physiology, University of Minnesota, Minneapolis, MN, USA; 3 Division of Diabetes, Endocrinology and Metabolism, Department of Medicine, University of Minnesota, Minneapolis, MN, USA

**Keywords:** cholesterol/cell and tissue, dietary fat, inflammation, lipase/hepatic, liver, NAFLD, lysosomal acid lipase, autophagy, immune infiltration, CE, cholesteryl ester, DE, differentially expressed, FPC, fructose, palmitate, and cholesterol, GO, Gene Ontology, IPA, ingenuity pathway analysis, iWAT, inguinal white adipose tissue, KC, Kupffer cell, LAL, lysosomal acid lipase, LD, lipid droplet, Mo/MF, monocyte-derived macrophage, NAFLD, nonalcoholic fatty liver disease, NK, natural killer, RNA-Seq, RNA sequencing, SFA, saturated FA, TAG, triacylglycerol, TEM, transmission electron microscope

## Abstract

Nonalcoholic fatty liver disease (NAFLD) is characterized by the accumulation of lipid droplets in hepatocytes. NAFLD development and progression is associated with an increase in hepatic cholesterol levels and decreased autophagy and lipophagy flux. Previous studies have shown that the expression of lysosomal acid lipase (LAL), encoded by the gene *LIPA*, which can hydrolyze both triglyceride and cholesteryl esters, is inversely correlated with the severity of NAFLD. In addition, ablation of LAL activity results in profound NAFLD. Based on this, we predicted that overexpressing *LIPA* in the livers of mice fed a Western diet would prevent the development of NAFLD. As expected, mice fed the Western diet exhibited numerous markers of NAFLD, including hepatomegaly, lipid accumulation, and inflammation. Unexpectedly, LAL overexpression did not attenuate steatosis and had only minor effects on neutral lipid composition. However, LAL overexpression exacerbated inflammatory gene expression and infiltration of immune cells in mice fed the Western diet. LAL overexpression also resulted in abnormal phagosome accumulation and lysosomal lipid accumulation depending upon the dietary treatment. Overall, we found that hepatic overexpression of LAL drove immune cell infiltration and inflammation and did not attenuate the development of NAFLD, suggesting that targeting LAL expression may not be a viable route to treat NAFLD in humans.

Lysosomal acid lipase (gene = *LIPA*, protein = LAL) is the only identified lipase in the lysosome and plays a key role in lipid metabolism. Triacylglycerols (TAGs) and cholesteryl esters (CEs) hydrolyzed in lysosomes are derived from two pathways: the endocytosis of circulating lipoproteins or the autophagic engulfment of lipid droplets (LDs), the latter of which is termed lipophagy (1, 2). Released FAs are largely effluxed via lysosomal exocytosis and reuptaken as needed (3), whereas cholesterol is transported out of the lysosome via the NPC1/2 proteins (4). Cholesterol produced via LAL plays a key role in regulating cellular cholesterol synthesis by inhibiting SREBP2 activation (5).

Deficiency or loss of *LIPA* leads to cholesteryl ester storage disease or Wolman disease, respectively (6). Cholesteryl ester storage disease is characterized by an enlarged fatty liver, hypercholesterolemia, and hypertriglyceridemia, whereas Wolman disease is fatal for humans early in life. Whole-body knockout of *Lipa* in mice results in accumulation of lipids in autophagosomes, severe hepatomegaly, fatty liver, and increased cholesterol synthesis (5, 7, 8). Furthermore, liver-specific KO promotes hepatic inflammation and lipid accumulation (9). However, hepatocyte-specific expression of human *LIPA* in a whole-body mouse KO model is able to rescue liver inflammation and ameliorate KO-induced effects in peripheral tissues (10), indicating that hepatic LAL expression is a key player in regulating whole-body metabolism.

Serum LAL activity has repeatedly been shown to decrease with the development of nonalcoholic fatty liver disease (NAFLD) (11, 12) and is correlated with reduced hepatic LAL activity (13). In addition to reduced LAL expression, autophagy as a whole is downregulated in response to various high-fat diet models (14, 15), in part because of inhibition of autophagic flux (16) and the prevention of acidification of lysosomes (17). Recent work also identifies lipophagy inhibition in clinical samples of NAFLD patients (18), leading some to suggest testing LAL replacement therapy as a potential treatment for diet-induced liver disease (19). A mouse model of LAL overexpression in adipose tissue prevented diet-induced weight gain and lowered circulating cholesterol (20). However, the potential impacts of liver-specific overexpression of LAL are unknown.

During the development of NAFLD, lipid accumulation leads to hepatocyte ballooning and apoptosis (21). In response, Kupffer cells (KCs), the resident macrophages in the liver, encircle the dying cells to degrade them, forming crown-like structures that accumulate cholesterol, resulting in a phenotypic change that recruits other immune cells and promotes inflammation (22, 23, 24). As this occurs, the KC pool is depleted and recruited monocytes fill the niche, but these monocyte-derived replacements are more inflammatory than KCs (25, 26). B cells are also recruited to the liver, where they drive inflammation and fibrogenesis (27). Multiple studies have demonstrated that preventing this recruitment of additional immune cells or removal of KCs slows the progression of NAFLD, indicating that immune cell infiltration is a key step in disease progression (27, 28, 29, 30, 31).

As the dysregulation of lipid metabolism is a driving force in NAFLD, we examined whether the hepatic overexpression of human *LIPA* could protect mice from developing NAFLD on a Western diet. In contrast to expectations, these studies revealed that LAL overexpression exacerbated NAFLD and the associated proinflammatory phenotype.

## Materials and methods

### AAV creation

pAAV.TBG.PI.eGFP.WPRE.bGH was a gift from James M. Wilson (Addgene; plasmid 105535). The *LIPA* gene, which codes for human LAL, was cloned into this plasmid to replace enhanced GFP. A similar system has previously been used to overexpress a transgene specifically in hepatocytes (32). The GFP and *LIPA* containing plasmids were packaged in AAV8 viral particles by the University of Minnesota Viral Vector and Cloning Core.

### Mice and diets

Seven-week-old male and female C57BL/6J mice were purchased from Jackson Laboratories and housed under controlled temperature (22°C) and lighting conditions (14:10 h light-dark cycle). To promote NAFLD, we used a high-fat diet with additional fructose, palmitate, and cholesterol (FPC) diet (33). Purified diet (TD.94048) and FPC diet (TD.190142) were purchased from Envigo (Indianapolis, IN). Mice fed the FPC diet also received fructose-glucose water (23.1 g/l d-fructose and 18.9 g/l d-glucose). Mice were acclimatized for 1 week prior to retroorbital injections with 5e^11^ viral copies/mouse of AAV harboring GFP or *LIPA*. After 1 week, dietary treatments were initiated for 16 weeks, and body weights were collected weekly. At week 17, mice were sacrificed following a 4 h fast, and tissues were collected. All animal protocols were approved by the University of Minnesota Institutional Animal Care and Use Committee.

### Lysosomal lipase activity assay

Biological samples (tissue lysate or serum) were added to 200 μM 4-methylumbelliferyl oleate in 100 mM acetate buffer, pH 4, with 1% Triton X-100. Fluorescence (excitation/emission = 320/460 nm) was measured for 3 h, and enzymatic activity was determined by comparing to a standard curve of 4-methylumbelliferone. Tissue lysate samples were normalized to total protein, and serum samples were normalized per volume.

### Western blotting

Liver, inguinal white adipose tissue (iWAT), and heart tissue were homogenized in RIPA buffer containing protease and phosphatase inhibitors and clarified. Protein concentration was determined via BCA assay (Thermo Fisher Scientific, Waltham, MA; 23225). Ponceau staining was used as a protein loading control, representative images are shown in figures, but the entire lane was used for quantification. Antibodies used were LAL (Origene, Rockville, MD; TA309730), CD45 (Abcam, Cambridge, United Kingdom; ab10558), F4/80 (Abcam, Cambridge, United Kingdom; ab74383), Phospho-ULK1 (Cell Signaling Technology, Danvers, MA; 6888), ULK1 (Cell Signaling Technology, Danvers, MA; 8054), Atg5 (Cell Signaling Technology, Danvers, MA; 12994), and LC3 (MBL International Corporation, Woburn, MA; PM036).

### Imaging and staining

Livers were preserved in formaldehyde and then paraffin embedded for H&E and PicroSirius Red staining. For Oil Red O staining, liver samples were preserved by OCT. All staining and sectioning were done by the University of Minnesota Histology Core. LD size analyses were performed using a CellProfiler pipeline to analyze H&E images and Oil Red O stain (34). Images were collected from liver sections from three mice per group, providing 2–6 images per liver for CellProfiler analyses. LDs with a diameter smaller than 2.75 μm were unable to be reliably quantified and were excluded as well as objects with a compactness >2 or eccentricity >0.94. PicroSirius Red stain was imaged using polarized light and quantified using a CellProfiler pipeline on images excluding central veins and portal triads.

### Lipid quantification

Total hepatic lipids were extracted using chloroform:methanol (2:1), dried under nitrogen gas, and quantified gravimetrically. Free cholesterol (for both males and females) and CE (for females) were determined enzymatically (35). TAG, CE, free cholesterol, and FA composition of TAG/CE for male mice were quantified via gas chromatographic analysis by the Vanderbilt University Hormone Assay and Analytical Services Core. Briefly, lipid classes are separated by thin layer chromatography using Silica Gel 60 A plates developed in petroleum ether, ethyl ether, acetic acid (80:20:1) and visualized by rhodamine 6G. TAG and CE are scraped from the plates and methylated using BF3/methanol. Gas chromatographic analysis is performed on an Agilent 7890A gas chromatograph equipped with flame ionization detectors and a capillary column (SP2380, 0.25 mm × 30 m, 0.20 μm film; Supelco, Bellefonte, PA).

### Serum metabolites

At sacrifice, blood was collected from the heart, and serum was isolated after centrifugation for 10 min at 5,000 *g*. TAG (SB-2100-430; Stanbio Laboratory, Boerne, TX), glucose (997-03001; Wako Diagnostics, Lexington, MA), ketone bodies (415-73301; Wako Diagnostics, Lexington, MA), NEFAs (999-34691; Wako Diagnostics, Lexington, MA), and cholesterol (999-02601; Wako Diagnostics, Lexington, MA) were measured using 96-well plate formats as per the manufacturer's instructions.

### RNA sequencing

RNA was isolated from liver tissue using a combined TRIzol (15596026; Invitrogen, Waltham, MA)/RNeasy kit (74004; Qiagen, Hilden, Germany) extraction and was submitted to the University of Minnesota Genomics Core for RNA sequencing (RNA-Seq) analysis. Briefly, total RNA samples were converted to Illumina sequencing libraries using Illumina's (San Diego, CA) TruSeq RNA Sample Preparation Kit (catalog no. RS-122-2001 or RS-122-2002) or stranded mRNA Sample Preparation Kit (catalog no. RS-122-2101). The libraries were then loaded onto the NovaSeq paired end flow cell, and clustering occured on board the instrument. Base call (.bcl) files for each cycle of sequencing were generated by Illumina Real-Time Analysis software. The base call files and run folders were streamed to servers maintained at the Minnesota Supercomputing Institute. Primary analysis and demultiplexing were performed using Illumina's bcl2fastq, version 2.20.

### Differential expression analysis of RNA-Seq

About 2 × 150 bp FastQ paired-end reads (n = 37.1 million average per sample) were trimmed using Trimmomatic (version 0.33) enabled with the optional “−q” command; 3 bp sliding-window trimming from 3′ end requiring minimum Q30. Quality control on raw sequence data for each sample was performed with FastQC. Read mapping was performed via Hisat2 (version 2.1.0) using the mouse genome (GRCm38) as reference. Gene quantification was done via Feature Counts for raw read counts. Differentially expressed (DE) genes were identified using the edgeR (negative binomial) feature in CLCGWB (Qiagen, Redwood City, CA) using raw read counts. We filtered the generated list based on a minimum 2× absolute fold change and false discovery rate-corrected *P* < 0.05. Data were analyzed through the use of ingenuity pathway analysis (IPA; QIAGEN, Inc, https://www.qiagenbioinformatics.com/products/ingenuitypathway-analysis) (36) and the Gene Ontology (GO) database (37).

### Quantitative PCR

RNA was isolated from liver tissue using a combined Trizol/RNeasy kit extraction, as previously described. cDNA was synthesized using Superscript Vilo cDNA Synthesis Kit (Invitrogen, Waltham, MA), and quantitative PCR was performed using SYBR Green Master Mix. All data shown were normalized to Cyc1 and TATA-binding protein expression using an extended ΔCT method (38).

### Immune cell isolation

Livers were collected, and at least 1 g was homogenized in RPMI media using a gentleMACS dissociator (Miltenyi Biotech, Bergisch Gladbach, Germany). A cell pellet was obtained by differential centrifugation using a 37.5% Percoll (Sigma-Aldrich, St. Louis, MO) gradient (39). Red blood cells were removed using a lysis buffer (BioLegend, San Diego, CA), and the cells were washed before counting with a Muse cell analyzer (Millipore Sigma, Burlington, MA).

### Mass cytometry

Immune cells were stained with 5 μM cisplatin to discriminate between viable and dead cells. Cisplatin staining was quenched with maxpar cell staining buffer, and nonspecific binding was blocked with TruStain FcX Plus (BioLegend, San Francisco, CA). Staining for cell surface markers was performed with 5 μg of metal-conjugated primary antibodies for 30 min at 4°C. Cells were then fixed with 1.6% formaldehyde (Thermo Fisher Scientific, Waltham, MA) and incubated with 0.5 μM intercalator solution in Fix and Perm buffer overnight. Cells were washed, resuspended in maxpar water, and data acquired on a CyTOF2 cytometer (DVS Sciences, Sunnyvale, CA). For intracellular staining, cells were fixed and permeabilized with maxpar buffers and stained with metal-conjugated intracellular antibodies. Mass cytometry data were analyzed using Cytobank (27). Immune cells were determined as a percent of total CD45+ cells, which were normalized to Western blots for CD45 to provide the total amount of each cell type per liver sample. All reagents for the mass cytometry analysis were from Fluidigm (San Francisco, CA) unless otherwise noted.

### Electron microscopy

Samples approximately 1–2 mm^3^ were initially placed in a fixative solution of 3% paraformaldehyde, 1.5% glutaraldehyde, and 2.5% sucrose in 0.1 M sodium cacodylate buffer with 5 mM calcium chloride and 5 mM magnesium chloride (pH 7.4) and kept at room temperature for 30 min, then stored for at least 24 h at 4°C. They were rinsed in buffer (10 min, 3×) and then placed in 1% osmium tetroxide and 0.1 M sodium cacodylate buffer (pH 7.4) overnight at 4°C. Specimens were rinsed in ultrapure water (NANOpure Infinity®; Barnstead/Thermo Fisher Scientific; Waltham, MA) (10 min, 3×), en bloc stained with 1% aqueous uranyl acetate for 2 h, and rinsed in ultrapure water (10 min, 3×). They were then dehydrated in an ethanol series (25%, 50%, 75%, 95% [2×] and 100% [3×]; 20 min for each change) and embedded in Embed 812 resin (Electron Microscopy Sciences, Hatfield, PA). Ultrathin sections 80–100 nm thick were cut on a Leica Ultracut UCT microtome using a diamond knife and collected on formvar/carbon-coated copper 200-mesh grids (Electron Microscopy Sciences). They were stained with 3% aqueous uranyl acetate for 20 min, rinsed in ultrapure water (10 s, 5×), stained with Sato's triple-lead stain (40) for 3 min, and rinsed in ultrapure water (10 s, 5×). Sections were examined with a JEOL JEM1400-Plus transmission electron microscope (TEM) operating at 60 kV. Images were recorded with a Maxim DL digital capture system. TEM image quantification is based on 4–7 images collected from three mice per group.

### Statistical analyses

All data are presented as individual values overlaid with means ± SEM. Differences between groups were determined using either a two-way ANOVA followed by Turkey's post hoc test or multiple unpaired *t*-tests. Statistical significance was declared at *P* < 0.05.

## Results

### FPC diet-induced hepatomegaly was unaffected by hepatic LAL overexpression

**Fig. 1:**
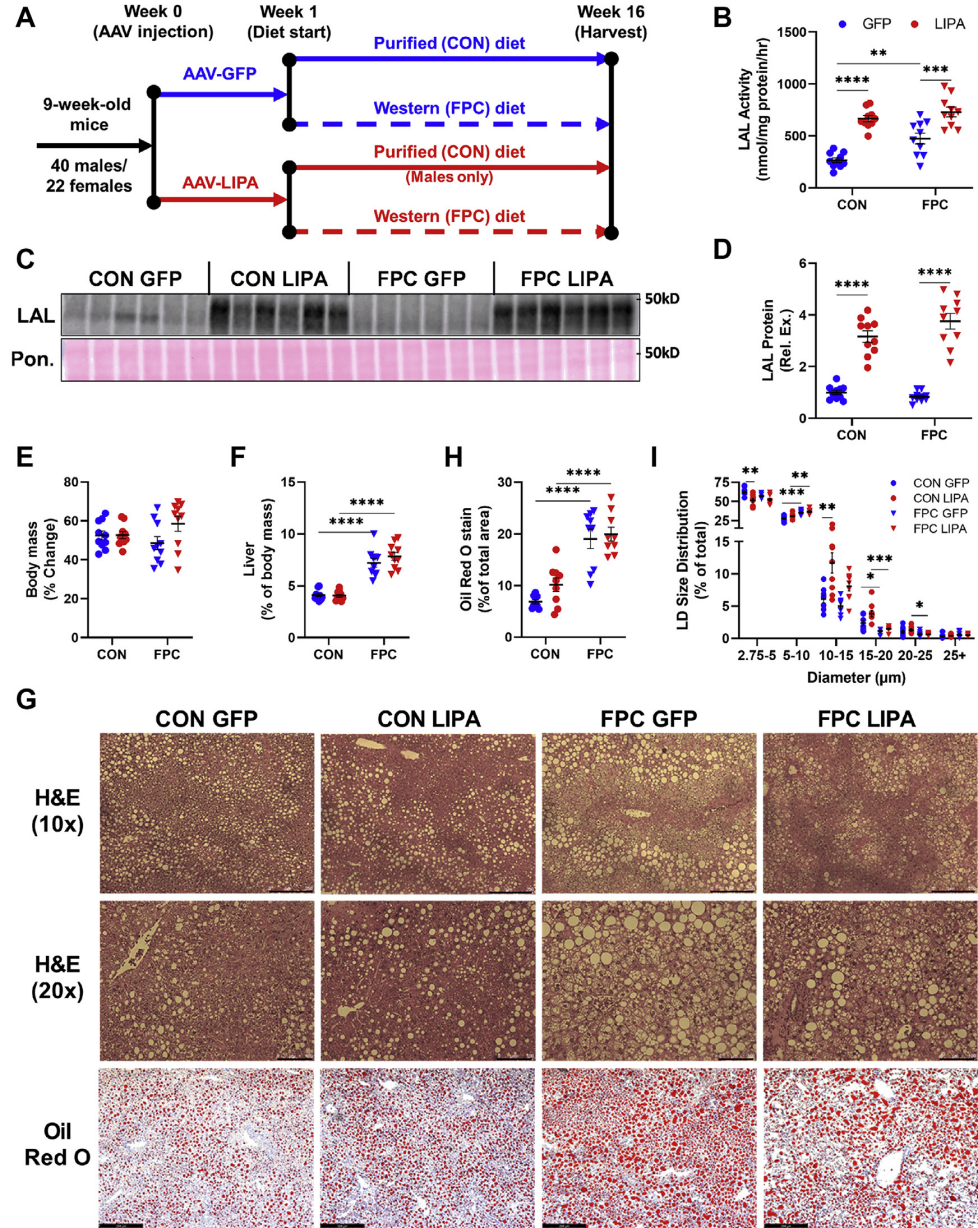
LAL overexpression alters hepatic LD size distribution. A: Experimental time line. B: Hepatic LAL activity measured via 4-MUO hydrolysis assay. C: Representative Western blot for hepatic LAL expression and (D) quantification. E: Percent body weight change from week 1 to week 16. F: Liver weights normalized to body weight. G: Representative images for H&E 10× (the scale bar represents 250 μm), 20× (the scale bar represents 100 μm) and Oil Red O (the scale bar represents 256 μm) staining for liver sections. H: Percent of total image area occupied by Oil Red O staining. I: Percent of hepatic LDs within each size group. All data shown are from male mice. Statistical comparisons are indicated by horizontal lines, and significant values are depicted as ∗*P* < 0.05, ∗∗*P* < 0.01, ∗∗∗*P* < 0.005, and ∗∗∗∗*P* < 0.001. 4-MUO, 4-methylumbelliferyl oleate.

**Supplemental Figure 1:**
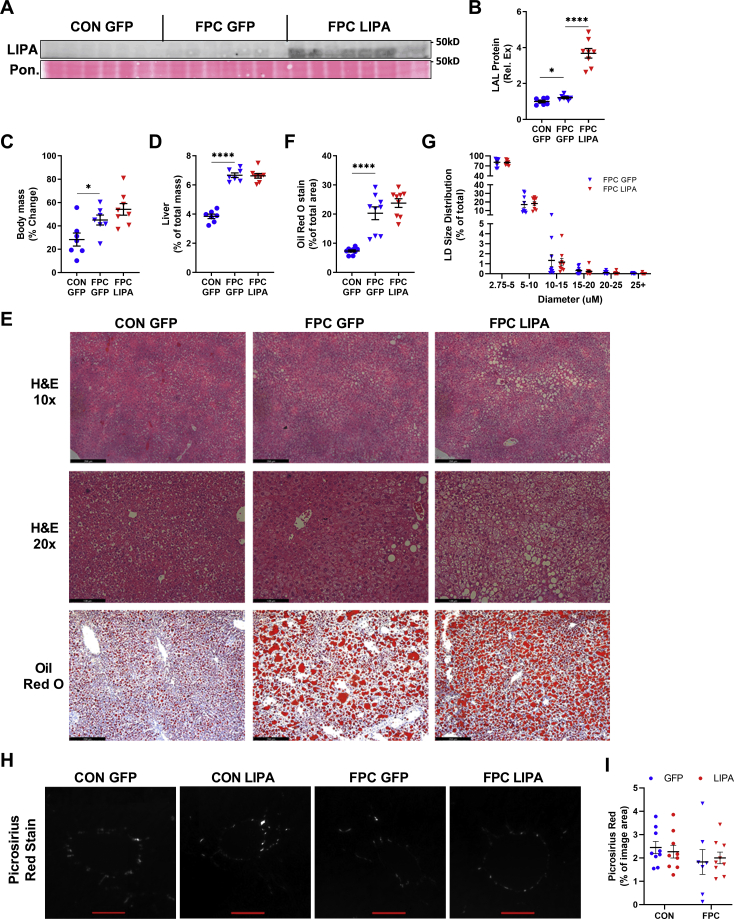
Data of female mice corresponding to Figure 1. A: Western blot for hepatic LAL expression and (B) quantification. C: Percent body weight change from week 1 to week 16. D: Liver weights normalized to the body weight. E: Representative images for H&E 10× (scale bar represents 250 μm) and 20× (scale bar represents 100 μm) and Oil red O (scale bar represents 256 μm) staining for liver sections. F: The percentage of the total image area occupied by Oil Red O staining. G: The percentage of hepatic LDs within each size group. I: Representative images for Picrosirius red staining containing portal triads. H: Quantification of Picrosirius red stain. Data in panels ∗*P* < 0.05, ∗∗∗∗*P* < 0.001. LAL, lysosomal acid lipase; LDs, lipid droplets.

**Supplemental Figure 2:**
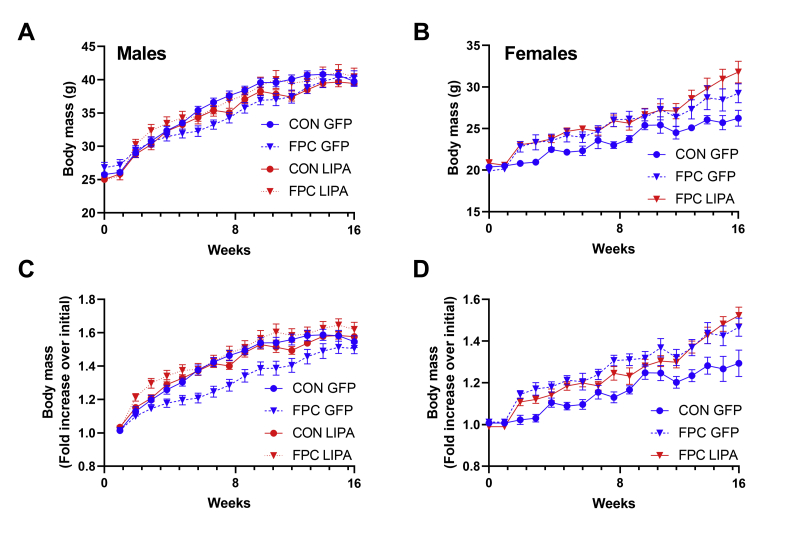
Change in the body mass. Weekly measures of the body mass for (A) male and (B) female mice. The body mass as a fold change compared to week 1 for (C) male and (D) female mice.

After 16 weeks on diet (Fig. 1A), hepatic protein lysates from mice that received the *LIPA* AAV had significantly higher hepatic LAL activity (Fig. 1B) and LAL expression (Fig. 1C, D and supplemental Fig. S1A, B) when compared with groups that received the GFP AAV. No significant changes were observed in body weight gain across the male groups (Fig. 1E), although the FPC diet did significantly increase body weight gain in females (supplemental Figs. S1C and S2). Regardless of sex and LAL expression, the livers of mice on the FPC diet weighed significantly more than mice on the purified diet (Fig. 1F and supplemental Fig. S1D), though no changes in fibrosis were observed (supplemental Fig. S1H, I). While the diet did not elicit the changes in body weight originally seen by Wang *et al.* (33), the diet promoted severe hepatomegaly, resulting in a nonobesogenic NAFLD model.

Histological staining of livers revealed variations in fat accumulation and LD populations across groups resulting from both LAL overexpression and the FPC diet (Fig. 1G–I and supplemental Fig. S1E–G). As expected, the FPC diet significantly increased the percent of total area stained by Oil Red O, though this was unaffected by LAL expression (Fig. 1H and supplemental Fig. S1F). After binning LDs by diameter and examining the percent of LDs in each size range (Fig. 1I and supplemental Fig. S1G), we observed a significant reduction in LDs with a diameter between 2.75 and 5 μm and an increase in LDs between 10 and 15 μm in the CON-*LIPA* group. While similar trends were present in FPC-fed mice, the significance was lost. This shift in LD size could indicate that LAL overexpression drives increased breakdown of smaller LDs, which are thought to be the preferred lipophagic substrates (41). These data show that while LAL overexpression did not prevent hepatomegaly or steatosis, it did have more subtle effects on hepatic LD size in mice on the control diet.

### LAL overexpression alters hepatic cholesterol stores

**Fig. 2:**
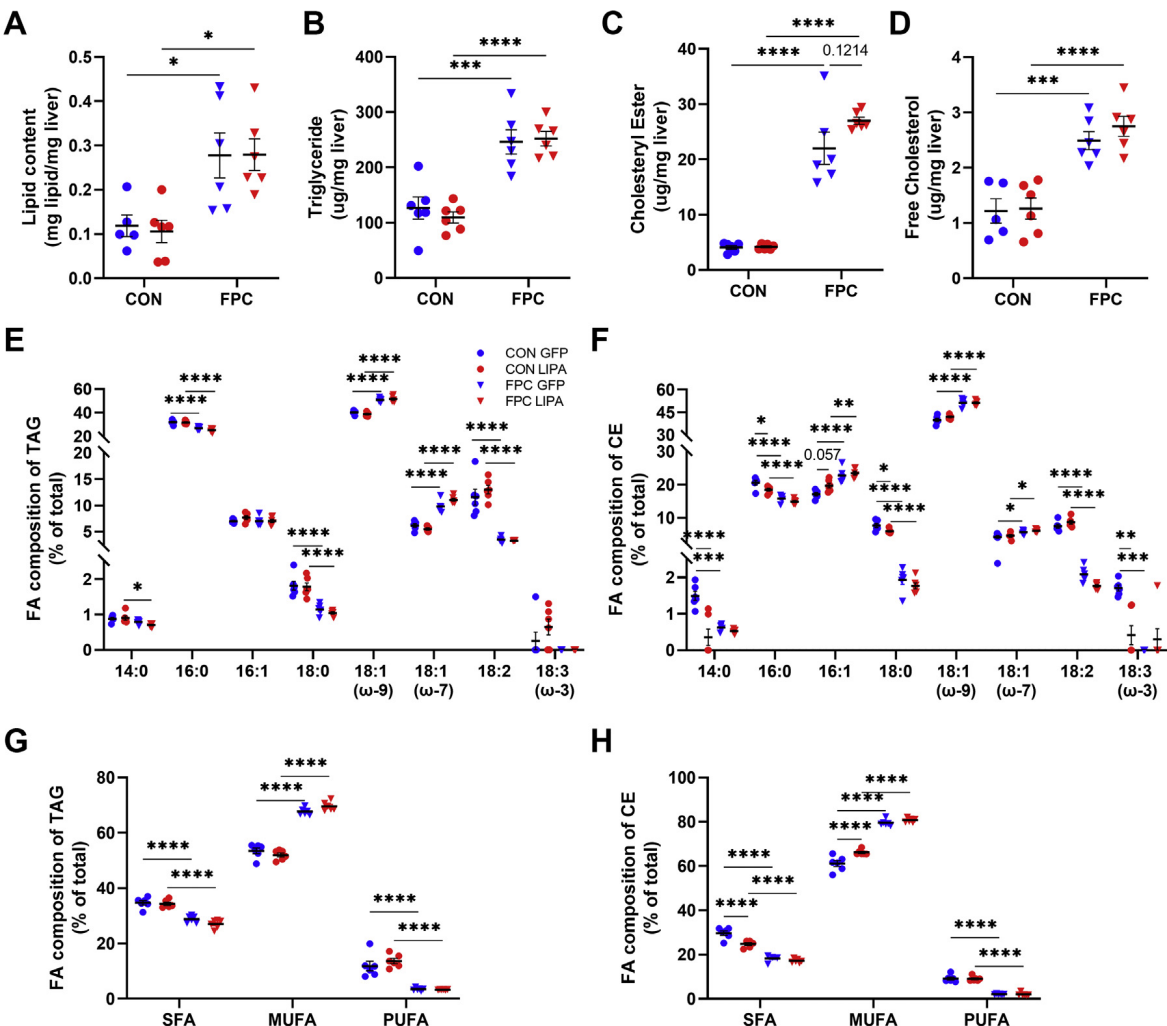
LAL overexpression modifies hepatic lipid species. A: Mass of all hepatic lipids per milligram of liver. B: Mass of triglycerides per milligram of liver. C: Mass of cholesterol per milligram of liver. D: Mass of cholesterol esters per milligram of liver. E: Percent composition of TAG pool categorized by acyl group. F: Percent composition of CE pool categorized by acyl group. G: Percent composition of the TAG pool categorized by FA type. H: Percent composition of the CE pool categorized by FA type. All data shown are from male mice. Statistical comparisons are indicated by horizontal lines, and significant values are depicted as ∗*P* < 0.05, ∗∗*P* < 0.01, ∗∗∗*P* < 0.005, and ∗∗∗∗*P* < 0.001.

**Supplemental Figure 3:**
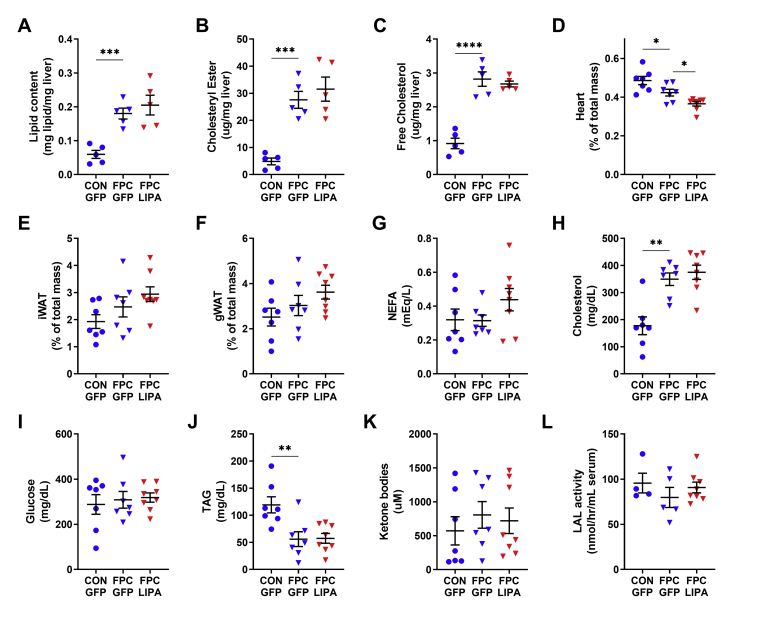
Hepatic lipids and peripheral analyses in female mice. A: Hepatic lipids per milligram of tissue. B: Mass of cholesteryl esters per milligram of the liver. C: Mass of free cholesterol per milligram of the liver. Weights for (D) the heart, (E) iWAT, and (F) gWAT normalized to the body weight. Serum levels for (G) NEFA, (H) cholesterol, (I) fasting glucose, (J) TAG, and (K) ketone bodies. L: Serum LAL activity measured via 4-MUO hydrolysis assay. All data shown are from female mice. Statistical comparisons are indicated by horizontal lines, and significant values are depicted as ∗*P* < 0.05, ∗∗*P* < 0.01, ∗∗∗*P* < 0.005, ∗∗∗∗*P* < 0.001. 4-MUO, 4-methylumbelliferyl oleate; iWAT, inguinal white adipose tissue; gWAT, gonadal white adipose tissue; LAL, lysosomal acid lipase; TAG, triacylglycerol.

Measurements of total hepatic lipids further confirm that the hepatomegaly is largely the result of lipid accumulation in mice on the FPC diet and was unaltered by LAL expression in both males (Fig. 2A) and females (supplemental Fig. S3A). Hepatic TAG, CE, and free cholesterol levels rose in mice receiving the FPC diet and were largely unaffected by LAL expression (Fig. 2B–D and supplemental Fig. S3B, C).

In addition to measuring total lipid levels, we analyzed the acyl chains present in both TAG and CE in the male mice (Fig. 2E, F). Significant changes were observed in the composition of TAG acyl groups in response to the FPC diet (Fig. 2E), with increases in 18:1 (ω-9) and 18:1 (ω-7) and decreases in 16:0, 18:2, and 18:0. However, no significant changes because of LAL expression were observed. In contrast, the acyl groups stored in CE were significantly altered in response to the FPC diet and to LAL overexpression alone (Fig. 2F). The FPC diet, regardless of LAL expression, significantly increased 16:1, 18:1 (ω-9), and 18:1 (ω-7), whereas 16:0, 18:0, and 18:2 were reduced. Notably, the pools of CE containing saturated FAs (SFAs) 14:0, 16:0, and 18:0 were all significantly reduced with LAL overexpression on the control diet, whereas there was a trend for increased 16:1. However, these LAL-driven changes in the CE pool disappeared on the FPC diet.

To better visualize the changes in FA partitioning, we sorted these data into SFA, MUFA, and PUFA for both the TAG and CE pools (Fig. 2G, H). The changes in mole percent of TAG composition in mice on FPC diet are similar to those seen in humans with NAFLD (Fig. 2G) (42), whereas LAL overexpression reduced the percent of CE containing SFA and increased MUFA on the control diet (Fig. 2H). As with the changes in LD size distribution, this change was lost in mice on the FPC diet.

### LAL overexpression and FPC diet promote changes in the heart and adipose depots

**Fig. 3:**
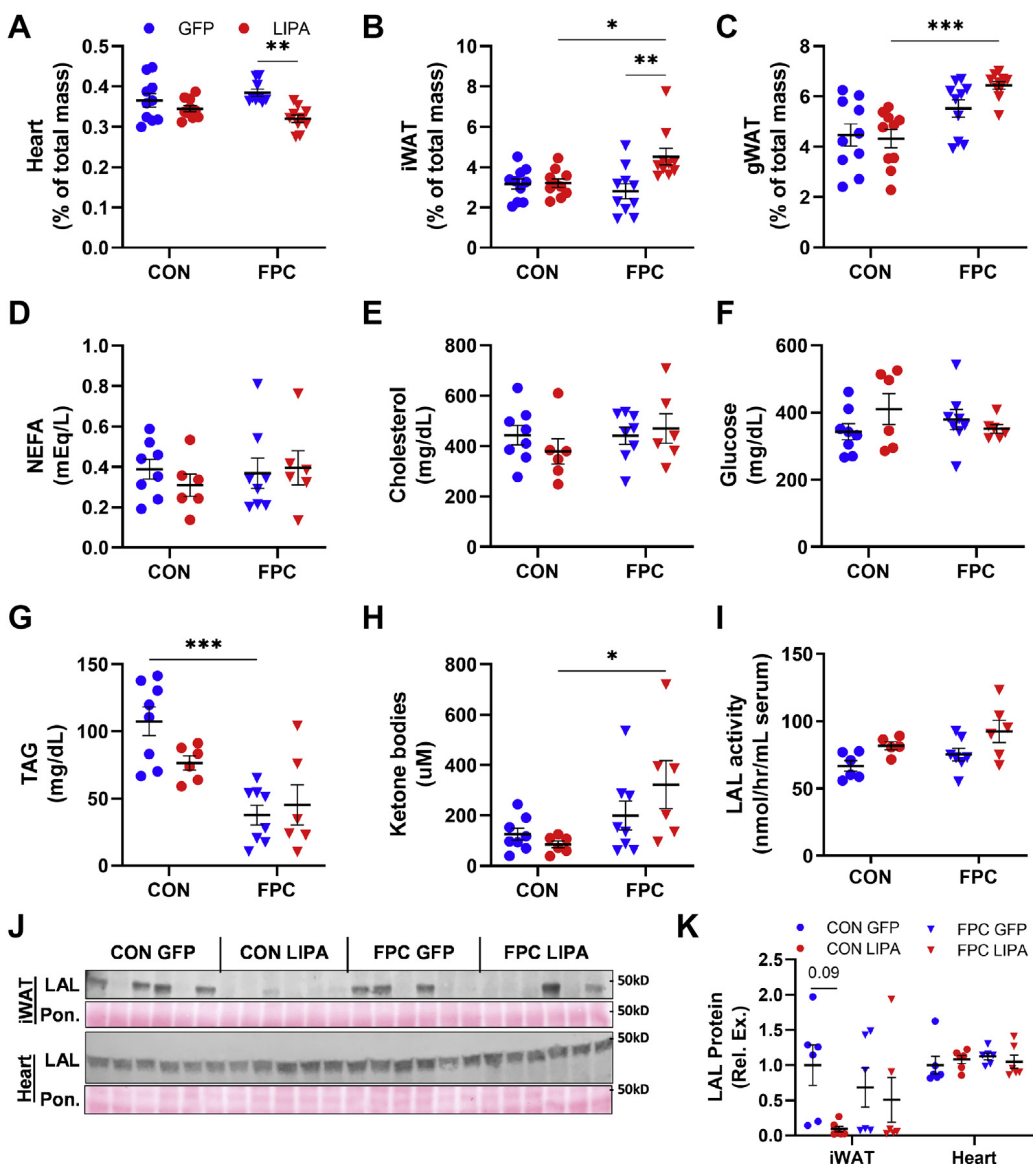
Whole-body treatment effects. Weights for (A) heart, (B) inguinal WAT, and (C) gonadal WAT normalized to body weight. Serum levels for (D) NEFA, (E) cholesterol, (F) fasting glucose, (G) TAG, and (H) ketone bodies. I: Serum LAL activity measured via 4-MUO hydrolysis assay. J: Western blots and (K) quantification for LAL expression in iWAT and heart tissue. All data shown are from male mice. Statistical comparisons are indicated by horizontal lines and significant values are depicted as ∗*P* < 0.05, ∗∗*P* < 0.01, ∗∗∗*P* < 0.005, and ∗∗∗∗*P* < 0.001. 4-MUO, 4-methylumbelliferyl oleate.

As liver LAL expression plays a key role in regulating metabolic changes throughout the body, we measured the weights of the heart, iWAT, and gonadal white adipose tissue, as well as the concentrations of several serum metabolites (Fig. 3 and supplemental Fig. S3D–K). Heart mass was significantly reduced in male and female mice on the FPC diet with LAL overexpression when compared with the FPC-GFP control (Fig. 3A and supplemental Fig. S3D). We were unable to identify any previously reported heart-specific phenotypes in *Lipa* KO models outside atherosclerosis-based studies, although polymorphisms in *LIPA* that increase expression are associated with impaired endothelial function and increased susceptibility to coronary artery disease (43).

iWAT depots from male mice in the FPC-*LIPA* group were significantly larger than those in the CON-*LIPA* and FPC-GFP groups (Fig. 3B). However, gonadal white adipose tissue depots did not exhibit the same trend, with only the *LIPA* groups showing a significant increase on the FPC diet (Fig. 3C). Despite differences in body weight, female mice did not have any significant differences in WAT depots (supplemental Fig. S3E, F).

No significant changes from LAL expression in serum NEFA, cholesterol, or fasting glucose levels were observed in male mice, whereas female mice had a significant increase in serum cholesterol in response to the FPC diet (Fig. 3D, F and supplemental Fig. 3G–I). In both sexes, serum TAG was significantly reduced in mice that were fed FPC diet, but no differences were observed in the LAL overexpression group (Fig. 3G and supplemental Fig. S3J). Ketone bodies were elevated in the LAL overexpression group in male mice fed the FPC diet (Fig. 3H) but were unchanged in females (supplemental Fig. S3K). As LAL is released with lysosomal exocytosis, we also measured serum LAL activity (Fig. 3I and supplemental Fig. S3L) to determine whether increased hepatic LAL impacted circulating LAL but found that it was not significantly changed across groups regardless of sex. Furthermore, LAL protein abundance in heart and iWAT tissue was measured in male mice (Fig. 3J, K). No significant differences were identified between groups, although there was large mouse to mouse variation in iWAT LAL expression levels. These data indicate that hepatic LAL overexpression does not result in increased LAL throughout the body and does not have a major effect on adiposity or serum metabolites.

### LAL overexpression alters the hepatic transcriptome

**Fig. 4:**
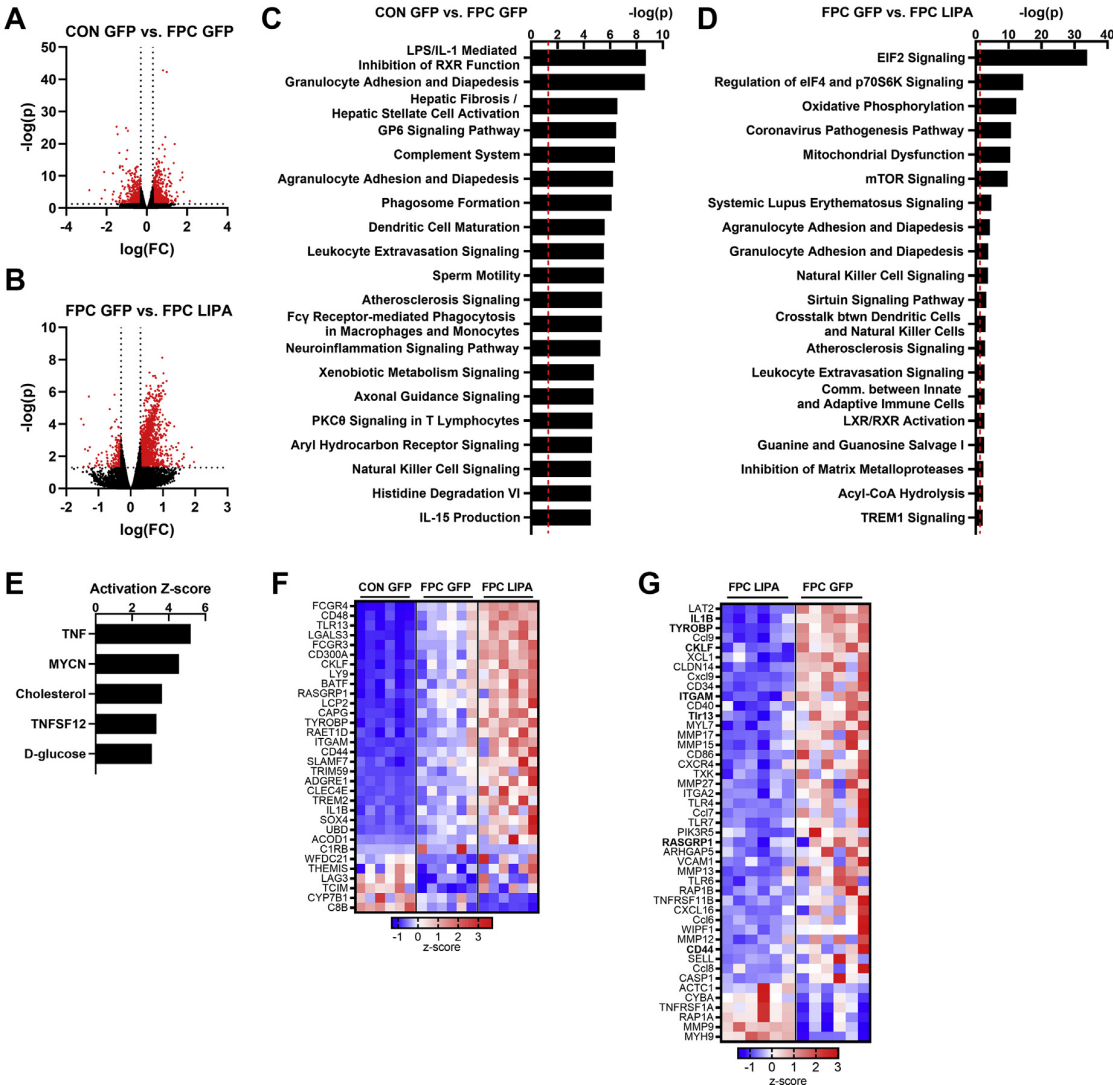
LAL overexpression altered the hepatic transcriptome. Volcano plots of differentially expressed genes when comparing (A) FPC GFP to CON GFP and (B) LIPA FPC to GFP FPC. Top canonical pathways predicted to be changed by IPA based on transcriptome changes between (C) FPC GFP to CON GFP and (D) LIPA FPC to GFP FPC. E: Top predicted upstream regulators of LIPA-induced gene changes. F: Heat map based on z-score for immune-related differentially expressed (DE) genes because of both FPC and LIPA effects. G: DE genes in the leukocyte extravasation, agranulocyte/granulocyte adhesion and diapedesis, and TREM1 signaling IPA groups. Bolded gene names in (F) are also present in (E). All data shown are from male livers. TREM1, triggering receptor expressed on myeloid cells-1.

**Supplemental Figure 4:**
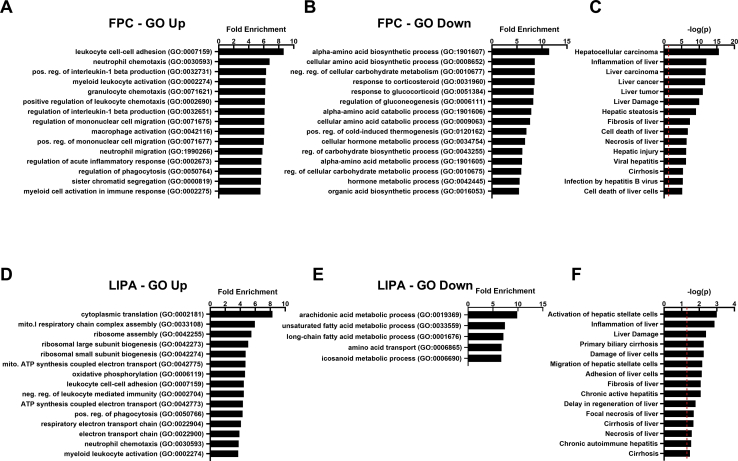
Gene ontology analysis of transcriptomic data. GO analysis of DE genes (A) increased and (B) decreased with FPC. C: Top terms returned using a liver-specific Tox Function analysis in IPA for the FPC DE genes. GO analysis of DE genes (D) increased and (E) decreased with LIPA. F: Top terms returned using a liver-specific Tox function analysis in IPA for the LIPA DE genes. DE, differentially expressed; FPC, fructose, palmitate, and cholesterol; GO, Gene Ontology; IPA, ingenuity pathway analysis.

To further investigate the effect of both the FPC diet and LAL overexpression on changes in the hepatic transcriptome, RNA-Seq analysis was conducted. Since our primary focus was on testing if LAL overexpression could remedy NAFLD, we conducted RNA-Seq analysis on livers from the CON-GFP, FPC-GFP, and FPC-*LIPA* groups to validate the effects of the FPC diet and to determine the effects of *LIPA* within the NAFLD-promoting FPC diet in male mice. There were 1,335 DE genes between the CON-GFP and FPC-GFP groups (Fig. 4A) and 1,879 between the FPC-GFP and FPC-*LIPA* groups (Fig. 4B). IPA revealed that the most significantly changed pathways as a result of the FPC diet were related to immune infiltration, hepatic fibrosis, stellate cell activation, and inflammation (Fig. 4C). These changes were largely expected as they are associated with the development of NAFLD. DE gene lists also underwent GO analysis, which revealed similar increases in immune pathways related to leukocyte activation and inflammation (supplemental Fig. S4A–C).

Using IPA, we identified the top pathways altered in response to LAL overexpression in the mice fed the FPC diet (Fig. 4D). While the most significant pathways were associated with protein translation and oxidative metabolism, many IPA pathways were related to immune cell infiltration and inflammation. This suggested that LAL overexpression resulted in substantial changes in the immune cell infiltration and inflammation, especially as these pathways were largely related to leukocyte extravasation/diapedesis and communication between immune cell populations. Similar inflammation-related pathways were identified in the GO analysis of DE genes (supplemental Fig. S4D–F). To determine potential drivers of the observed changes, we performed an IPA upstream regulator analysis and found cholesterol to be one of the top endogenous chemicals predicted to be upregulated (Fig. 4E), suggesting that alternate pathways of cholesterol signaling that we were unable to capture may be responsible for some of the observed phenotype.

While similar pathways were significantly changed with the FPC diet or LAL overexpression, only 186 genes were significantly changed under both the FPC and *LIPA* conditions. Of these genes, 32 are involved in the immune response (GO: 0002376) and have been plotted as a heat map to show the changes in this gene list across groups (Fig. 4F). Most of the genes in this group increase in expression with FPC and *LIPA*, potentially reflecting an exacerbation of hepatic inflammation.

To further examine the changes in inflammation that resulted from LAL overexpression, we generated a heat map of the DE genes between FPC-GFP and FPC-*LIPA* groups that were enriched in the diapedesis and lymphocyte extravasation (Fig. 4G). We found that LAL overexpression resulted in increased expression of genes encoding for immune activation, proinflammatory cytokines, chemokines, and toll-like receptors. Taken together, these changes suggest that LAL overexpression is significantly driving the proinflammatory response associated with the FPC diet.

### LAL overexpression promotes hepatic immune infiltration and inflammation

**Fig. 5:**
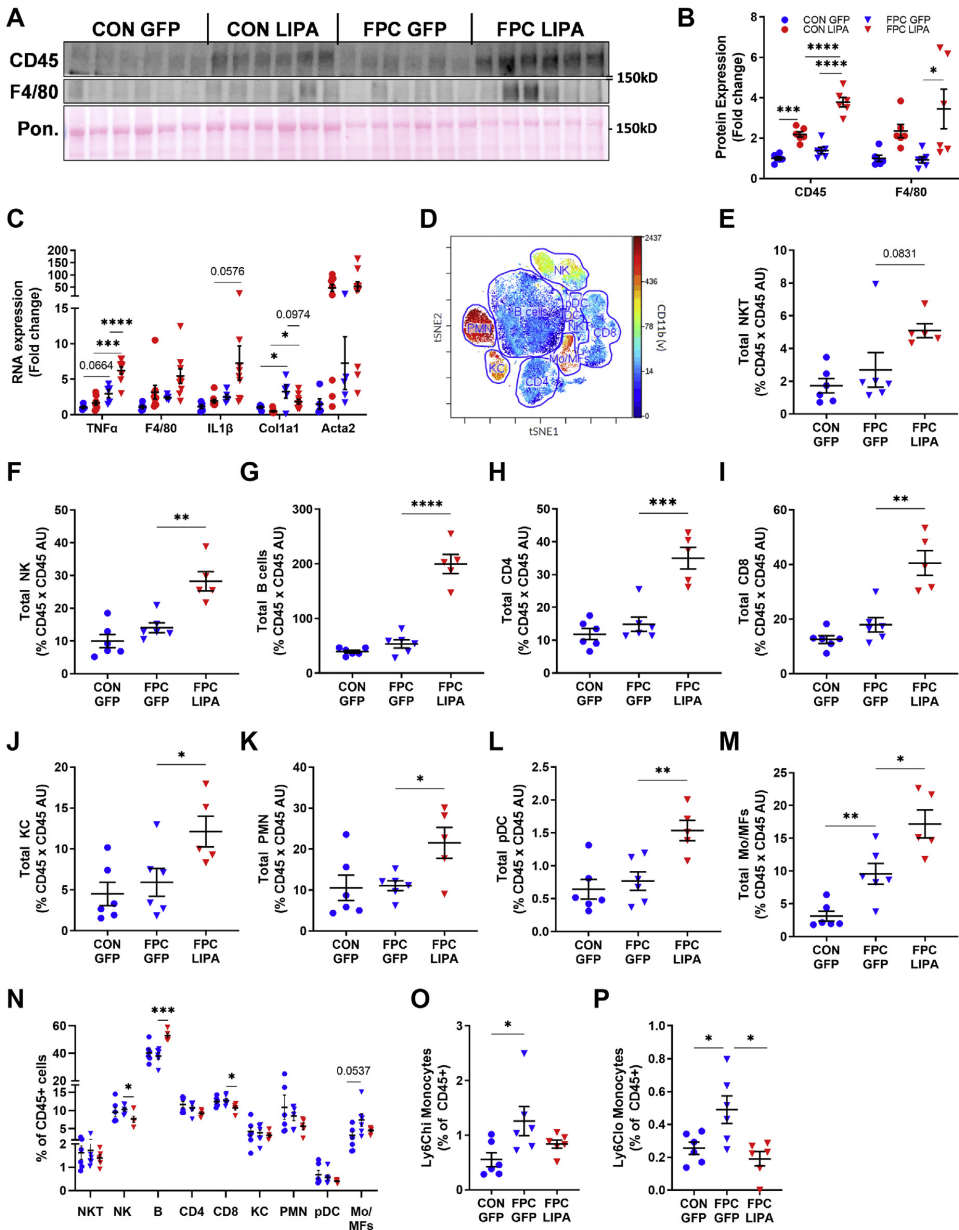
LAL overexpression promoted immune cell infiltration and inflammation. A: Westerns for immune markers in hepatic protein lysates and (B) quantification. C: Hepatic RNA levels of immune and fibrotic markers determined by quantitative PCR. D: Representative plot of immune cell populations measured via CyTOF. Mass cytometry analyses normalized to CD45 protein levels for (E) NKT cells, (F) NK cells, (G) B cells, (H) CD4+ cells, (I) CD8+ cells, (J) KCs, (K) polymorphonuclear cells (PMNs), (L) plasmacytoid dendritic cell (pDC), and (M) Mo/MFs. N: Immune cell populations as a percent of the total CD45+ cell pool. Ly6Chi (O) and Ly6Clo (P) monocytes as a percentage of the total CD45+ pool. All data shown are from male mice. Statistical comparisons are indicated by horizontal lines, and significant values are depicted as ∗*P* < 0.05, ∗∗*P* < 0.01, ∗∗∗*P* < 0.005, and ∗∗∗∗*P* < 0.001.

**Supplemental Figure 5:**
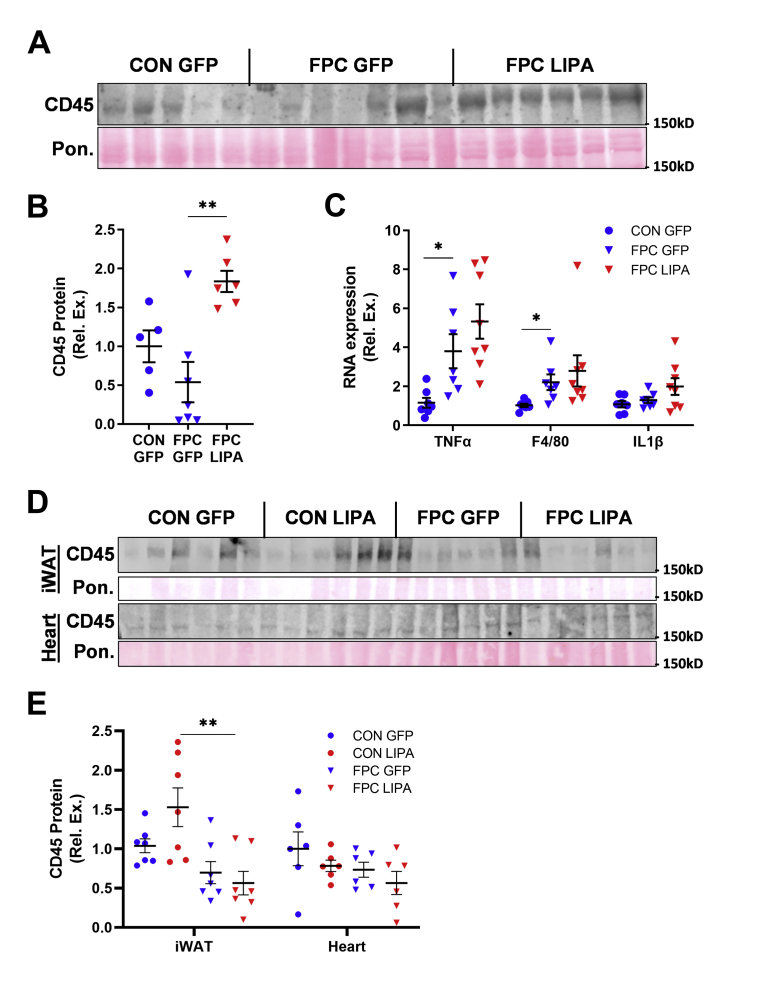
Immune measurements in female and systemic infiltration in male mice. A: Western blot for CD45 in hepatic protein lysates and (B) quantification. C: Hepatic RNA levels of immune markers determined by qPCR. D: Representative Western blots and (E) quantification for CD45 expression in iWAT and heart tissue. Data in panels A–D are from female mice, and those in panels D and E are from male mice. Statistical comparisons are indicated by horizontal lines, and significant values are depicted as ∗*P* < 0.05, ∗∗*P* < 0.01. iWAT, inguinal white adipose tissue.

To better understand how LAL overexpression instigates hepatic inflammation, we characterized the immune cell infiltrates in the liver. First, we measured the protein content and mRNA expression of several inflammatory markers. Western blots were performed for CD45 and F4/80, which are a leukocyte and a macrophage marker, respectively (Fig. 5A, B). LAL overexpression increased the expression of CD45 in the livers of mice on both diets and F4/80 in the FPC-fed group. Female mice showed a similar increase in CD45 expression in hepatic lysate (supplemental Fig. S5A, B). Next, we performed quantitative PCR for a subset of inflammatory and fibrotic genes (*Tnfα, F4/80, Il1β, Col1a1,* and *Acta2*) including tissue from CON-*LIPA* group (Fig. 5C and supplemental Fig. S5C). Both *Tnfα* and *Il1β* were highly expressed in response to LAL overexpression in male mice fed the FPC diet (Fig. 5C). However, this effect was not seen in female mice, which had increased *Tnfα* and *F4/80* mRNA levels in response to the FPC diet, not LAL overexpression (supplemental Fig. S5C). Male mice showed significant increases in *Col1a1* mRNA in response to the FPC diet, but there was no significant change in *Acta2* expression because of high mouse to mouse variability (Fig. 5C).

We then isolated the immune cells from the livers and performed CyTOF to identify the major immune subsets (Fig. 5D). The abundance of natural killer (NK) T cells (Fig. 5E) was not affected by dietary treatment but trended to increase with LAL expression. The amounts of NK (Fig. 5F) cells, B cells (Fig. 5G), CD4+ T cells (Fig. 5H), CD8+ T cells (Fig. 5I), KCs (Fig. 5J), polymorphonuclear cells (Fig. 5K), and plasmacytoid dendritic cells (Fig. 5L) present in the liver were increased with LAL overexpression but unaltered on the FPC diet alone. Monocytes and monocyte-derived macrophages (Mo/MFs) were significantly increased by the FPC diet and LAL overexpression (Fig. 5M). Overall, these findings suggest that LAL overexpression results in a substantial accumulation of all major immune cell subsets. As the infiltration of monocyte and Mo/MF population is associated with proinflammatory signaling, these data suggest that LAL overexpression promotes inflammation through an increase in monocyte recruitment, as suggested by our IPA analysis.

Examination of the relative abundance of immune cell subsets showed that LAL overexpression increased the percent of B cells, whereas the frequency of NK and CD8+ T cells decreased (Fig. 5N). The FPC diet elicited similar trends in the Mo/MF frequency and total abundance. To further characterize the Mo/MF population, we used an alternative gating strategy to quantify Ly6Chi (Fig. 5O) and Ly6Clo (Fig. 5P) monocytes. These monocytes are associated with inflammatory and restorative phenotypes, respectively (44). Both these populations increased as a percentage of total CD45+ cells with the FPC diet, but only the restorative Ly6Clo population decreased with LAL overexpression. In addition, this increased immune cell infiltration was not observed in iWAT or heart tissue (supplemental Fig. S5D, E), suggesting that the liver is the primary site of inflammation. Collectively, these data show that LAL overexpression enhanced the proinflammatory liver phenotype of mice on the FPC diet.

### LAL overexpression drives autolysosome accumulation and lysosomal LDs

**Fig. 6:**
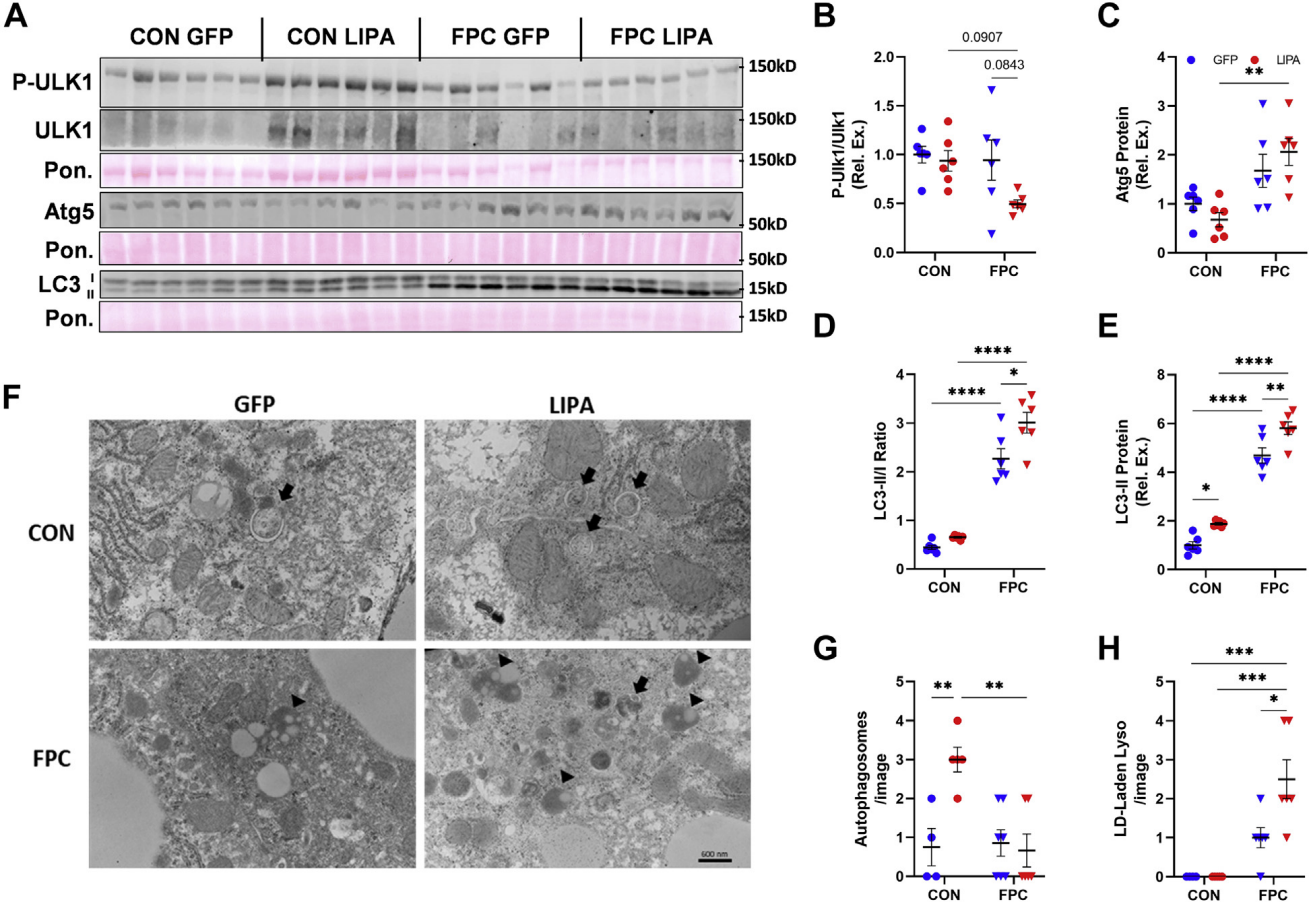
LAL overexpression alters autophagosome abundance. A: Western blots probing for phospho (Ser757)-Ulk1, total Ulk1, LC3, and Atg5 expression. B: Quantification of the fold change in Ulk1 phosphorylation. C: Quantification of Atg5 expression. LC3 expression quantified as both (D) LC3-II/I ratio and (E) LC3-II expression. F: Representative TEM images (the scale bar represents 600 nm) displaying autophagosomes (arrow) and lipid-laden lysosomes (arrowhead). Numbers of (G) autophagosomes and (H) lipid-laden lysosomes per image (n = 2–3 images/mouse, three mice/group). All data shown are from male mice. Statistical comparisons are indicated by horizontal lines, and significant values are depicted as ∗*P* < 0.05, ∗∗*P* < 0.01, ∗∗∗*P* < 0.005, and ∗∗∗∗*P* < 0.001.

**Supplemental Figure 6:**
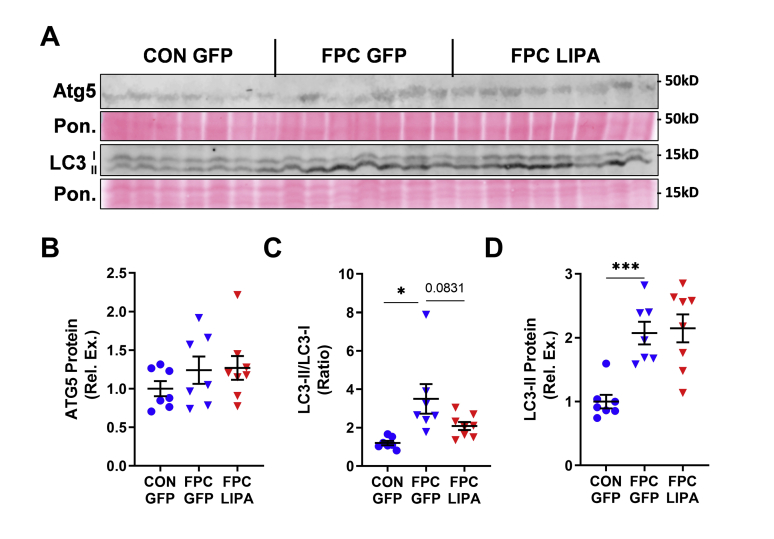
Autophagic measurements in female mice. A: Autophagic proteins measured via Western blot. B: Quantified Atg5 expression. LC3 expression quantified as both (C) LC3-II/I ratio and (D) LC3-II. All data shown are from female mice. Statistical comparisons are indicated by horizontal lines, and significant values are depicted as ∗*P* < 0.05, ∗∗∗*P* < 0.005.

As NAFLD is often associated with the disruption of autophagy, we assessed livers for changes in protein markers of autophagy (Fig. 6A and supplemental Fig. S6A). Upstream signaling was assessed by measuring Ulk1 phosphorylation by mammalian target of rapamycin at Ser757, which inhibits the initiation of autophagy. Phospho-Ulk1 trended toward being reduced in the FPC-*LIPA* group when compared with every other group (Fig. 6B). Atg5 and LC3 lipidation were measured as indicators of autophagosome formation and abundance. The FPC diet resulted in a significant increase in Atg5 expression in male mice (Fig. 6C and supplemental Fig. S6B) and LC3II/I ratio in both sexes (Fig. 6D and supplemental Fig. S6C), indicating an increase in the abundance of autophagic machinery. LAL overexpression on the FPC diet significantly increased LC3 lipidation, represented by the LC3II/I ratio, in male mice, whereas female mice trended toward a reduction, suggesting there may be sex-specific differences in autophagy that require further examination. As the FPC diet did not alter upstream activation as measured by phospho-Ulk1, this suggests that autophagy is inhibited at a downstream step, which matches previous studies showing that a high-fat diet inhibits autophagic flux (45).

Here, we also quantified LC3-II alone as an approximation of mature autophagosomes and early autolysosomes and observed that, regardless of dietary conditions, LAL overexpression significantly increases LC3-II expression in male mice (Fig. 6E). Female mice do not exhibit a difference in LC3-II expression with LAL overexpression (supplemental Fig. S6D). The data from male mice suggest that LAL is either promoting autophagosome formation or inhibiting LC3 removal and breakdown from the autophagosome membrane after lysosomal fusion. However, as we only see a trend in phospho-Ulk1 in the FPC-*LIPA* group, it seems less likely that LAL is regulating autophagosome formation and more likely that lysosomal lipids are accumulating and stalling autophagy.

To further investigate the impact of LAL overexpression, TEM of male liver samples was performed to identify changes in autophagic machinery (Fig. 6F). We observed an increase in autophagosomes in the CON-*LIPA* group compared with the CON-GFP group, again suggesting that LAL overexpression either drives autophagosome formation or prevents breakdown (Fig. 6G). In addition, autophagosomes were largely absent from FPC livers. In both FPC groups, we identified an increase in LD-laden lysosomes, with the FPC-*LIPA* group containing more (Fig. 6H). These data suggest that LAL overexpression, despite increasing LAL enzymatic activity, negatively impacts lysosomal lipid degradation especially under FPC feeding conditions.

## Discussion

In this study, we sought to determine whether the overexpression of LAL attenuated the development of fatty liver disease in mice on a Western diet. However, LAL overexpression did not prevent the accumulation of hepatic TAG or cholesterol because of the FPC diet, although LAL alone drove changes in LD size and the composition of CE populations. This was surprising as the importance of lysosomal-derived sterols in regulating cholesterol homeostasis and hepatic cholesterol levels has previously been established, although the involvement of SREBP2 in this system is controversial (9, 46). However, these studies were based on loss of function, in contrast to the overexpression model used in the current study. Previous research has shown that SREBP2 regulation is activated when endoplasmic reticulum cholesterol dips below 5% of the total lipid content (47). Thus, it is possible that the control GFP groups would not be below this threshold, so overexpressing LAL would not make a difference in SREBP2 activation.

Unexpectedly, the overexpression of LAL alone increases autophagosome and lysosomal accumulation to some degree. However, this increase was not associated with reduced hepatomegaly and lipid accumulation in the liver, suggesting that despite the increases in autophagic measures, promoting LAL expression is not sufficient to prevent NAFLD. The reduced number of small LDs coupled with the increase in autophagic measures further supports the assertion that lipophagic degradation preferentially targets small LDs (41). The mechanism by which LAL overexpression alters autophagy remains to be elucidated. Previous studies have shown that rescuing lysosomal pH, which was increased with steatosis, restored autophagy flux (48). This leads us to question whether therapies aimed at restoring lysosomal pH may be more viable than enhancing LAL abundance or activity.

The alterations in PUFA and MUFA content of CE suggest that LAL overexpression preferentially changes CE composition, especially when compared with the lack of changes seen in the TAG pool. It remains unclear whether there is a change in synthesis or breakdown that is altering the CE pool. There is no evidence suggesting that the process by which LDs are engulfed by autophagosomes or lysosomes is a selective process. LAL enzymatic selectivity for different substrates has only been biochemically using a fluorescent assay, although no FA longer than oleate was used (49). As such, we cannot rule out that LAL has a specific preference for degrading CEs containing SFAs. It is worth noting that the changes resulting from LAL overexpression shift the CE pool to resemble more closely that of mice on the FPC diet. However, the CE pool is much smaller than the TAG pool, so it is difficult to establish whether these changes hold biological significance.

While the total amount of all immune cells was increased on the FPC diet with LAL expression, B cells showed one of the highest increases in response to LAL overexpression. While the development of hepatic inflammation during NAFLD development and progression involves numerous immune and parenchymal cell types, B cells are becoming increasingly recognized for their role in NAFLD-induced inflammation (27). In addition to a general increase in immune cells likely promoting inflammation, LAL overexpression also reduced proresolution Ly6Clo monocytes, which may have further contributed to the state of elevated hepatic inflammation. These changes in hepatic immune cells mirror the robust increase in numerous proinflammatory pathways revealed from the RNA-Seq analysis, which reinforces that LAL overexpression promotes an inflammatory phenotype.

Our data strongly indicate that LAL overexpression drives hepatic inflammation; however, we have not identified a specific mechanism at work. Previous studies have shown that disruption of hepatocyte LAL expression drives immune infiltration as a result of excess cholesterol (9), and that the expression of LAL in macrophages (50) or hepatocytes (10) in *Lipa*-deficient mice reduces this inflammation. As hepatic cholesterol levels do not change with LAL overexpression, we do not think that cholesterol accumulation is responsible for the observed inflammation. However, as we measured total hepatic cholesterol, our data do not rule out the possibility of localized or cell type-specific fluctuations in cholesterol driving the observed inflammation.

Overall, we have shown that hepatic LAL overexpression is unable to prevent the development of NAFLD on a Western diet, providing evidence against using recombinant LAL as a potential treatment for NAFLD. We also show that overexpression drives inflammation and promotes a large influx of immune cells into the liver. This is contrary to overexpression of *LIPA* in adipose tissue, which showed reduced inflammation and no changes in autophagy (20) suggesting that the liver may be particularly sensitive to alterations of lysosomal lipid degradation and signaling.

## Data availability

RNA-Seq data are available in the NCBI Gene Expression Omnibus database (GSE180377).

## Supplemental data

This article contains supplemental data.

## Conflict of interest

The authors declare that they have no conflicts of interest with the contents of this article.
